# Hydromagnetic Flow and Heat Transfer over a Porous Oscillating Stretching Surface in a Viscoelastic Fluid with Porous Medium

**DOI:** 10.1371/journal.pone.0144299

**Published:** 2015-12-14

**Authors:** Sami Ullah Khan, Nasir Ali, Zaheer Abbas

**Affiliations:** 1 Department of Mathematics and Statistics, International Islamic University, Islamabad 44000, Pakistan; 2 Department of Mathematics, The Islamia University of Bahawalpur, Bahawalpur 63100, Pakistan; North China Electric Power University, CHINA

## Abstract

An analysis is carried out to study the heat transfer in unsteady two-dimensional boundary layer flow of a magnetohydrodynamics (MHD) second grade fluid over a porous oscillating stretching surface embedded in porous medium. The flow is induced due to infinite elastic sheet which is stretched periodically. With the help of dimensionless variables, the governing flow equations are reduced to a system of non-linear partial differential equations. This system has been solved numerically using the finite difference scheme, in which a coordinate transformation is used to transform the semi-infinite physical space to a bounded computational domain. The influence of the involved parameters on the flow, the temperature distribution, the skin-friction coefficient and the local Nusselt number is shown and discussed in detail. The study reveals that an oscillatory sheet embedded in a fluid-saturated porous medium generates oscillatory motion in the fluid. The amplitude and phase of oscillations depends on the rheology of the fluid as well as on the other parameters coming through imposed boundary conditions, inclusion of body force term and permeability of the porous medium. It is found that amplitude of flow velocity increases with increasing viscoelastic and mass suction/injection parameters. However, it decreases with increasing the strength of the applied magnetic field. Moreover, the temperature of fluid is a decreasing function of viscoelastic parameter, mass suction/injection parameter and Prandtl number.

## Introduction

Many fluids in industry and technology do not obey the Newton's law of viscosity and are usually classified as a non-Newtonian fluids. For example, blood, yogurt, ketchup, shampoo, polymer melts and greases exhibit complicated relationship between the shear stress and rate of strain. The boundary layer flow and heat transfer analysis of these fluids on a continuously moving surface has wide range of applications in engineering and industrial processes, for example, manufacturing of plastic sheets, artificial fibers and polymeric sheets, plastic foam processing, extrusion of polymer sheet from a die, heat materials travelling between a feed roll and many others. After the work of Sakiadis [1], many researchers studied the various aspects of flow and heat transfer characteristics of non-Newtonian fluids with/without magnetic field over a stretching surface. Some important contributions were due to Rajagopal et al. [2], Dundapat and Gupta [3], McLeod and Rajagopal [4], Rollins and Vajravelu [5], Cortell [6, 7], Nazer et al. [8], Ishak et al. [9], Hayat et al. [10], Khan et al. [11], Mohanty et al. [12], Tripathy et al. [13], Baag et al. [14], Mishra et al. [15–17] and many references therein. In above mentioned investigations the stretching velocity of sheet is linearly proportional to the distance along the flow direction. To the best of our knowledge, Wang [18] was first who studied the viscous flow due to oscillatory stretching surface which is stretched back and forth in its own plane. Abbas et al. [19] extended the problem of Wang [18] by including the heat transfer effects in the presence of velocity and thermal slip conditions. In another attempt, Abbas et al. [20] analyzed the boundary layer flow of a second grade fluid due to oscillatory stretching surface in the presence of magnetic field. The non-linear partial differential equation in [20] was solved both analytically and numerically. For analytical treatment homotopy analysis method was applied while numerical solution was based on finite difference technique. Zheng et al. [21] used homotopy analysis method to discuss Soret and Dufour effects in two-dimensional boundary layer flow of viscous fluid over an oscillatory stretching sheet. Ali et al. [22] studied the effects of heat transfer in hydromagnetic flow of a Jeffrey fluid over an oscillatory stretching sheet by using homotopy analysis method and finite difference scheme. In another paper, Ali et al [23] discussed the effects of heat source/sink and thermal radiation on unsteady flow of third grade fluid over an oscillatory stretching surface with convective boundary conditions. The effects of heat transfer on unsteady oblique stagnation-point flow of viscous fluid over an oscillating plate have been discussed by Javed et al. [24].Gul et al. [25] discussed an unsteady MHD thin film flow of an Oldroyd-B fluid over an oscillating inclined belt by using optimal homotopyasymptoticmethod and homotopyperturbationmethod. In another attempt, Gul et al. [26] discussed the effects of heat transfer in thin film flow of second grade fluid over a vertical oscillating belt. Recently, Sheikh et al. [27] discussed thermophoresis and heat generation/absorption effects on unsteady flow of viscous fluid over an oscillatory stretching sheet.

In this paper, we are interested to investigate hydromagnetic flow and heat transfer over a porous oscillating stretching sheet embedded in a porous medium. The rheological properties of the fluid are captured by using the constitutive equation of second grade fluid. This study extends the analysis of Abbas et al. [20], where a rigid sheet is embedded in a clear medium and heat transfer analysis is lacking. The governing equations are transformed into the set of non-linear partial differential equations by employing appropriate transformations. A numerical solution based on finite difference method is obtained. The effects of flow parameters on fluid velocity, temperature field, skin friction coefficient and local Nusselt number are shown through graphs and tables.

## Flow Analysis

**Fig 1:**
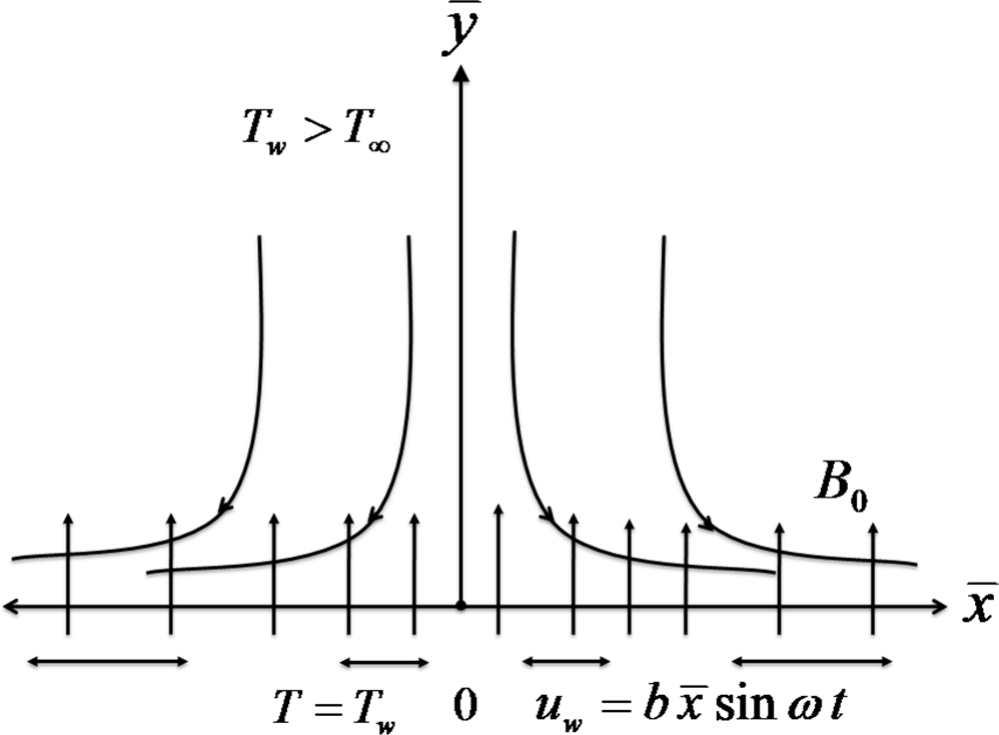
Geometry of the problem.

We consider the unsteady and two-dimensional magnetohydrodynamics (MHD) flow of incompressible viscoelastic fluid (second grade fluid) through a porous medium over a porous oscillatory stretching sheet coinciding with plane $\bar{y}=0$ (see Fig 1). A magnetic field of strength *B*_0_ is applied in the direction perpendicular to the sheet. In our case, we are only interested in studying the effects of applied magnetic field on the fluid motion and not the vice versa. In this case the diffusion of magnetic field is important and thus the magnetic susceptibility is large which results in a small magnetic Reynolds number. In the small magnetic Reynolds number limit, the induced magnetic field and electric current can be neglected in comparison with the applied magnetic field and current density, respectively. In nutshell, our analysis is based on the assumption of small magnetic susceptibility and this assumption is not violated when strength of applied magnetic field is large. For detail the readers are referred to the book of Davidson [28].The temperature of the sheet is maintained at a constant value *T*_*w*_ and far away from the sheet the temperature of ambient fluid is *T*_∞_, where *T*_*w*_ > *T*_∞_. Under these assumptions along with the boundary layer approximations and neglecting viscous dissipation, the governing equations based on conservation of mass, momentum and energy in presence of body force are:

$$\frac{\partial u}{\partial \bar{x}}+\frac{\partial v}{\partial \bar{y}}=0,$$ (1)

$$\frac{\partial u}{\partial t}+u\frac{\partial u}{\partial \bar{x}}+v\frac{\partial u}{\partial \bar{y}}=\nu \frac{\partial^{2}u}{\partial {\bar{y}}^{2}}+\frac{k_{0}}{\rho }\left[\frac{\partial^{3}u}{\partial t\partial {\bar{y}}^{2}}+\frac{\partial }{\partial \bar{x}}\left(u\frac{\partial^{2}u}{\partial {\bar{y}}^{2}}\right)+\frac{\partial u}{\partial \bar{y}}\frac{\partial^{2}v}{\partial {\bar{y}}^{2}}+v\frac{\partial^{3}u}{\partial {\bar{y}}^{3}}\right]-\frac{\sigma B_{0}^{2}}{\rho }u-\frac{\nu \phi }{k}u,$$ (2)

$$\rho c_{p}\left(\frac{\partial T}{\partial t}+u\frac{\partial T}{\partial \bar{x}}+v\frac{\partial T}{\partial \bar{y}}\right)=k_{1}\frac{\partial^{2}T}{\partial {\bar{y}}^{2}}\;,$$ (3)

where *u* and *v* are velocity component along $\bar{x}-$ and $\bar{y}-$ directions, respectively, *ν* is the kinematic viscosity, *t* is the time, *ρ* is the density, *k*_0_ is the normal stress coefficient, *σ* is electric conductivity, *ϕ* is porosity parameter, *k* is permeability of porous medium, *c*_*p*_ is the specific heat at constant pressure, *k*_1_ is the thermal conductivity and *T* is the temperature of fluid.

The flow is subjected to the following boundary conditions

$$u=u_{\omega }=b\bar{x}\;\text{sin}\;\omega t,\;v=-v_{w},\;T=T_{w}\;\text{at}\;\bar{y}=0,\;t>0,$$ (4)

$$u=0,\;\frac{\partial u}{\partial \bar{y}}=0,\;T\to T_{\infty }\;\text{as}\;\bar{y}\to \infty ,$$ (5)

where *v*_*w*_ is the wall mass transfer velocity with (*v*_*w*_ > 0) corresponds to the mass suction velocity and (*v*_*w*_ < 0) corresponds to the mass injection velocity, *ω* is the oscillation frequency and *b* is the stretching rate. The second condition in (5) is augmented condition since the flow is in unbounded domain.

Let us introduce the following dimensionless variables

$$\begin{array}{l} y=\sqrt{\frac{b}{\nu }}\bar{y},\;\tau =t\omega ,\;u=b\bar{x}f_{y}\left(y,\tau \right),\;v=-\sqrt{\nu b}f\left(y,\tau \right),\\ \theta (y,\tau )=\frac{T-T_{\infty }}{T_{w}-T_{\infty }}\;, \end{array}$$ (6)

where subscript denotes differentiation with respect to the indicated variables. With the help of Eq (6), the continuity equation is satisfied identically and Eqs (2) and (3) give

$$Sf_{y\tau }+f_{y}^{2}-ff_{yy}+\beta f_{y}=f_{yyy}+K\left(Sf_{yyy\tau }+2f_{y}f_{yyy}-f_{yy}^{2}-ff_{yyyy}\right),$$ (7)

$$\theta_{yy}+\mathrm{Pr}\left(f\theta_{y}-S\theta_{\tau }\right)=0.$$ (8)

The boundary conditions (4) and (5) take the following form

$$f_{y}\left(0,\tau \right)=\text{sin}\;\tau ,\;f\left(0,\tau \right)=\gamma ,\;\theta (0,\tau )=1,$$ (9)

$$f_{y}\left(\infty ,\tau \right)=0,\;f_{yy}\left(\infty ,\tau \right)=0,\;\theta (\infty ,\tau )=0.$$ (10)

In above equations $\gamma =v_{w}/\sqrt{\nu b}$ is the dimensionless mass suction/injection parameter, *K* = *bk*_0_ / *νρ* in the non-dimensional viscoelastic parameter, *S* ≡ *ω* / *b* is the ratio of the oscillation frequency of the sheet to its stretching rate, Pr = *μc*_*p*_ / *k* is the Prandtl number and $\beta =\sigma B_{0}^{2}/\rho b+\upsilon \phi /kb$ is a combined parameter due to magnetic field and the permeability of the porous medium. For non-conducting fluids, *σ* = 0 and as a result *β* = *υϕ* / *kb* corresponds to the classical permeability parameter and, by taking *k* → ∞, as a result $M=\sigma B_{0}^{2}\;/\;\rho b$ corresponds to the classical Hartmann number.

The physical quantities of interest are the skin-friction coefficient *C*_*f*_ and the local Nusselt number *Nu*_*x*_, which are defined as

$$C_{f}=\frac{\tau_{w}}{\rho u_{w}^{2}}\;,\;Nu_{x}=\frac{\bar{x}q_{w}}{k\left(T_{w}-T_{\infty }\right)}\;,$$ (11)

where *τ*_*w*_ and *q*_*w*_ are the shear stress and heat flux at wall, respectively, which are defined as

$$\tau_{w}=\mu {\left(\frac{\partial u}{\partial \bar{y}}\right)}_{\bar{y}=0}+k_{0}{\left(\frac{\partial^{2}u}{\partial t\partial \bar{y}}+u\frac{\partial^{2}u}{\partial \bar{x}\partial \bar{y}}+v\frac{\partial^{2}u}{\partial {\bar{y}}^{2}}-2\frac{\partial u}{\partial \bar{y}}\frac{\partial v}{\partial \bar{y}}\right)}_{\bar{y}=0},\;q_{w}=-k{\left(\frac{\partial T}{\partial \bar{y}}\right)}_{\bar{y}=0}.$$ (12)

In view of Eqs (6) and (12), Eq (11) gives

$${\text{Re}}_{x}^{1/2}C_{f}={\left[f_{yy}+K\left(3f_{y}f_{yy}+Sf_{yy\tau }-ff_{yyy}\right)\right]}_{y=0}\;,\;{\text{Re}}_{x}^{-1/2}Nu_{x}=-\theta_{y}\left(0,\tau \right),$$ (13)

where ${\text{Re}}_{x}=u_{w}\bar{x}/\nu$ is the local Reynold number.

## Direct Numerical Solution of the Problem

In this section, we present the solution of nonlinear boundary value problem consisting of Eqs (7) and (8) with boundary conditions (9) and (10) using finite difference method. For this purpose, we use the coordinate transformation *η* = 1 / *y*+1 to transform the semi-infinite physical domain *y* ∈ [0, ∞) to finite calculation domain *η* ∈ [0,1]. Employing this transformation, we get

$$y=\frac{1}{\eta }-1,\;\frac{\partial }{\partial y}=-\eta^{2}\frac{\partial }{\partial \eta },\;\frac{\partial^{2}}{\partial y^{2}}=\eta^{4}\frac{\partial^{2}}{\partial \eta^{2}}+2\eta^{3}\frac{\partial }{\partial \eta },\;\frac{\partial^{2}}{\partial y\partial \tau }=-\eta^{2}\frac{\partial^{2}}{\partial \eta \partial \tau },$$

$$\frac{\partial^{3}}{\partial y^{3}}=-\eta^{6}\frac{\partial^{3}}{\partial \eta^{3}}-6\eta^{5}\frac{\partial^{2}}{\partial \eta^{2}}-6\eta^{4}\frac{\partial }{\partial \eta }\;,\;\frac{\partial^{4}}{\partial y^{3}\partial \tau }=-\eta^{6}\frac{\partial^{4}}{\partial \eta^{3}\partial \tau }-6\eta^{5}\frac{\partial^{3}}{\partial \eta^{2}\partial \tau }-6\eta^{4}\frac{\partial^{2}}{\partial \eta \partial \tau }\;,$$

$$\frac{\partial^{4}}{\partial y^{4}}=\eta^{8}\frac{\partial^{4}}{\partial \eta^{4}}+12\eta^{7}\frac{\partial^{3}}{\partial \eta^{3}}+36\eta^{6}\frac{\partial^{2}}{\partial \eta^{2}}+24\eta^{5}\frac{\partial }{\partial \eta }.$$

Using above transformations in (7) and (8), we have the following equations and boundary conditions

$$\begin{array}{c} S\left(1-6K\eta^{2}\right)\frac{\partial^{2}f}{\partial \tau \partial \eta }-SK\eta^{4}\frac{\partial^{4}f}{\partial \eta^{3}\partial \tau }-6SK\eta^{3}\frac{\partial^{3}f}{\partial \eta^{2}\partial \tau }=\left(\eta^{2}-8K\eta^{4}\right){\left(\frac{\partial f}{\partial \eta }\right)}^{2}+(6\eta^{2}-\beta +24Kf\eta^{3}-2f\eta )\frac{\partial f}{\partial \eta }\\ \;+\eta^{4}\frac{\partial^{3}f}{\partial \eta^{3}}+\left(6\eta^{3}-f\eta^{2}+36Kf\eta^{4}\right)\frac{\partial^{2}f}{\partial \eta^{2}}-8K\eta^{5}\frac{\partial f}{\partial \eta }\frac{\partial^{2}f}{\partial \eta^{2}}\\ \;+K\eta^{6}{\left(\frac{\partial^{2}f}{\partial \eta^{2}}\right)}^{2}-2K\eta^{6}\frac{\partial f}{\partial \eta }\frac{\partial^{3}f}{\partial \eta^{3}}+12K\eta^{5}f\frac{\partial^{3}f}{\partial \eta^{3}}+K\eta^{6}f\frac{\partial^{4}f}{\partial \eta^{4}}\;, \end{array}$$ (14)

$$\eta^{4}\frac{\partial^{2}\theta }{\partial \eta^{2}}+2\eta^{3}\frac{\partial \theta }{\partial \eta }-\text{Pr}\left(f\eta^{2}\frac{\partial \theta }{\partial \eta }+S\frac{\partial \theta }{\partial \tau }\right)=0,$$ (15)

$$f_{\eta }=0,\;f_{\eta \eta }=0,\;\theta =0\;\text{at}\;\eta =0,$$ (16)

$$f=\gamma ,\;f_{\eta }=-\text{sin}\;\tau ,\;\text{at}\;\theta =1\;\eta =1,$$ (17)

Now we discretize Eqs (14) and (15) for *L* uniformly distributed discrete points in *η* = (*η*_1_, *η*_2_,……, *η*_{*L*}_) ∈ (0, 1) with a space grid size of Δ*η* = 1 / (*L* + 1) and the time level *t* = (*t* ^1^, *t* ^2^,….). Hence the discrete values $\left(f_{1}^{n},f_{2}^{n},\;\ldots \ldots ,f_{L}^{n}\right)$ and $\left(\theta_{1}^{n},\;\theta_{2}^{n},\;\ldots ..,\theta_{L}^{n}\right)$ at these grid point for time levels *t* ^*n*^ = *n*Δ*t* (Δ*t* is the time step size) can be numerically solved together with boundary conditions at *η* = *η*_0_ = 0 and *η* = *η*_{*L*+1}_ = 1, (16) and (17), as the initial conditions are given. We start our simulations from a motionless velocity field and a uniform temperature distribution equal to temperature at infinity as

f(η,τ=0)=0andθ(η,τ=0)=0. (18)

The oscillatory motion of the sheet with a temperature *T*_*w*_(*θ* = 1) is suddenly set from *τ* = 0 at *η* = 1 (*y* = 0). We will see that this periodic motion will be immediately reached within first five period. We construct a semi-infinite time difference for *f* and *θ*, respectively, and make sure that only linear equations for the new time step (*n* + 1) need to be solved:

$$\begin{array}{l} \;S(1-6K\eta^{2})\frac{1}{\Delta t}\left(\frac{\partial f^{\left(n+1\right)}}{\partial \eta }-\frac{\partial f^{\left(n\right)}}{\partial \eta }\right)-SK\eta^{4}\frac{1}{\Delta t}\left(\frac{\partial^{3}f^{\left(n+1\right)}}{\partial \eta^{3}}-\frac{\partial^{3}f^{\left(n\right)}}{\partial \eta^{3}}\right)-6SK\eta^{3}\frac{1}{\Delta t}\left(\frac{\partial^{2}f^{\left(n+1\right)}}{\partial \eta^{2}}-\frac{\partial^{2}f^{\left(n\right)}}{\partial \eta^{2}}\right)\\ =\left(\eta^{2}-8K\eta^{4}\right){\left(\frac{\partial f^{\left(n\right)}}{\partial \eta }\right)}^{2}+\left(6\eta^{2}-\beta \right)\frac{\partial f^{\left(n+1\right)}}{\partial \eta }+(24K\eta^{3}-2\eta )f^{\left(n\right)}\frac{\partial f^{\left(n\right)}}{\partial \eta }+6\eta^{3}\frac{\partial^{2}f^{\left(n+1\right)}}{\partial \eta^{2}}\\ \;+\left(36K\eta^{4}-\eta^{2}\right)f^{\left(n\right)}\frac{\partial^{2}f^{\left(n\right)}}{\partial \eta^{2}}-8K\eta^{5}\frac{\partial f^{\left(n\right)}}{\partial \eta }\frac{\partial^{2}f^{\left(n\right)}}{\partial \eta^{2}}+K\eta^{6}{\left(\frac{\partial^{2}f^{\left(n\right)}}{\partial \eta^{2}}\right)}^{2}+\eta^{4}\frac{\partial^{3}f^{\left(n+1\right)}}{\partial \eta^{3}}\\ \;-2K\eta^{6}\frac{\partial f^{\left(n\right)}}{\partial \eta }\frac{\partial^{3}f^{\left(n\right)}}{\partial \eta^{3}}+12K\eta^{5}f^{\left(n\right)}\frac{\partial^{3}f^{\left(n\right)}}{\partial \eta^{3}}+K\eta^{6}f^{\left(n\right)}\frac{\partial^{4}f^{\left(n\right)}}{\partial \eta^{4}}. \end{array}$$ (19)

$$S\;\text{Pr}\frac{\left(\theta^{\left(n+1\right)}-\theta^{\left(n\right)}\right)}{\Delta t}=\eta^{4}\frac{\partial^{2}\theta^{\left(n+1\right)}}{\partial \eta^{2}}+2\eta^{3}\frac{\partial \theta^{\left(n+1\right)}}{\partial \eta }-\text{Pr}\;f^{\left(n\right)}\eta^{2}\frac{\partial \theta^{\left(n+1\right)}}{\partial \eta }.$$ (20)

It should be noted that other different choices of time differences are also possible. By means of the finite difference method we can obtain two systems of linear equations for $f_{i}^{\left(n+1\right)}$ and $\theta_{i}^{\left(n+1\right)}$ *i* = (1, 2,…., *L*) at the time step (*n* + 1), which can be solved, e.g. by the Guassian elimination. This method has already been implemented by several authors to simulate other similar flows [29–31].

## Results and Discussion

The system of non-linear partial differential equations consisting of Eqs (14) and (15) with boundary conditions (16) and (17) has been solved numerically using finite difference scheme to compute the velocity and temperature profiles. The transverse distributions and time-series for velocity and temperature fields in the first five periods *τ* ∈ [0, 10*π*] are plotted to analyze the influence of the various parameters, for example, the viscoelastic parameter *K*, the mass suction/injection parameter *γ*, the rate of frequency to stretching rate *S*, combined parameter of magnetic field and permeability of porous medium *β* and the Prandtl number Pr. Furthermore, we compute and show the values of the skin friction coefficient ${\text{Re}}_{x}^{1/2}C_{f}$ and the local Nusselt number ${\text{Re}}_{x}^{-1/2}Nu_{x}$ for different parameters both graphically and in tabular form.

**Fig 2:**
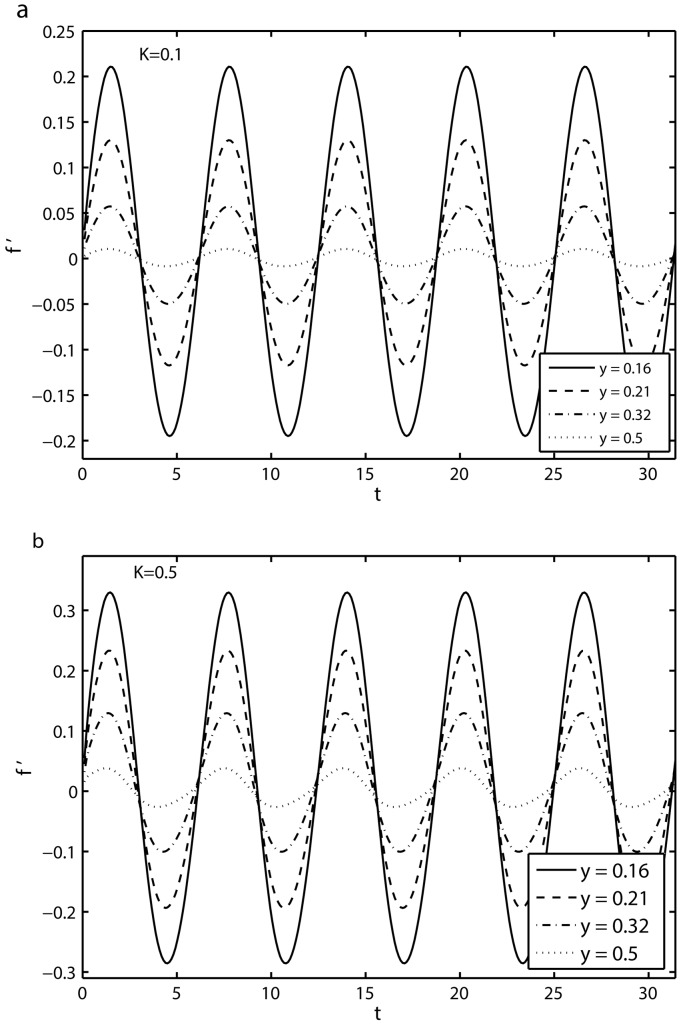
Time-series of the velocity *f*′ at the four different distances from the sheet for the time period *τ* ∈ [0, 10*π*] with *S* = 2, *β* = 10, *γ* = 0.5: (*a*) *K* = 0.1 and (*b*) *K* = 0.5.

Fig 2 shows the time-series of the velocity component *f*′ in the first five periods *τ* ∈ [0, 10*π*] for four different values of *y*(which correspond to different distances from the sheet) for *S* = 2, *β* = 10, *γ* = 0.5 and *K* = 0.1. It is evident from Fig 2(A) that with the increase of distance from the oscillatory sheet, the amplitude of flow velocity decreases. It is further noted that far away from the surface, the amplitude of the flow motion is almost negligible. We observe a similar phenomenon from Fig 2(B) for *K* = 0.5. However, for *K* = 0.5 the amplitude of the flow motion is large in comparison with the corresponding amplitude for *K* = 0.1.

**Fig 3:**
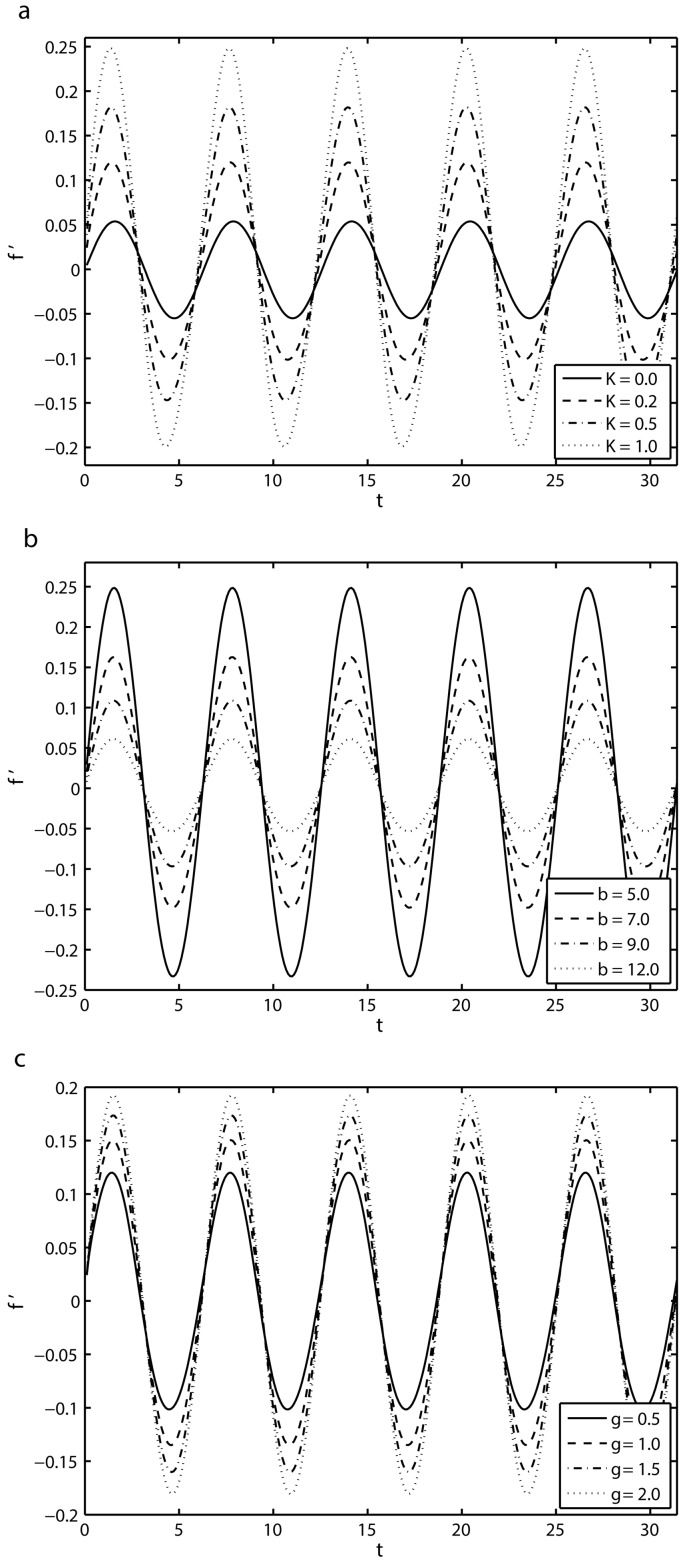
Time-series of velocity *f*′ in the first five periods *τ* ∈ [0, 10*π*] at a fixed distance from the sheet, *y* = 0.25: (*a*) effects of viscoelastic parameter *K* with *S* = 2, *β* = 10 and *γ* = 0.5 (*b*) effects of *β* with *S* = 1, *K* = 0.1, *γ* = 0.5 and (*c*) effects of *γ* with *S* = 2, *K* = 0.2 and *β* = 10.

Fig 3A–3C illustrates the influence of the viscoelastic parameter *K*, the combined parameter of magnetic field and permeability of porous medium *β* and the mass suction/injection parameter *γ* on the time-series of the velocity component *f*′ at a fixed distance *y* = 0.25 from the sheet, respectively. Fig 3(A) shows the effect of the viscoelastic parameter *K* on the time-series of the velocity profile *f*′ for *S* = 2, *β* = 10 and *γ* = 0.5. It is seen that the amplitude of the flow motion increases by increasing the value of *K* due to the increased effective viscosity and a phase shift occurs which increases with *K*. Fig 3(B) displays the effect of *β* on the time-series of the velocity component *f*′. It is found that the amplitude of the flow motion decreases with the increase of *β*. In fact, an increase in *β* corresponds to either an increase in strength of the applied magnetic field or a decrease in the permeability of the porous medium. In either case, the resistance to flow is increased and as a result of that the amplitude of flow velocity is suppressed. Fig 3(C) shows the time-series of velocity field *f*′ for different values of the mass suction/injectionparameter *γ*. It is evident from this figure that the amplitude of the flow motion increases for the large values *γ*. It is also noted that a phase shift occurs which increases with the increase of *γ*.

**Fig 4:**
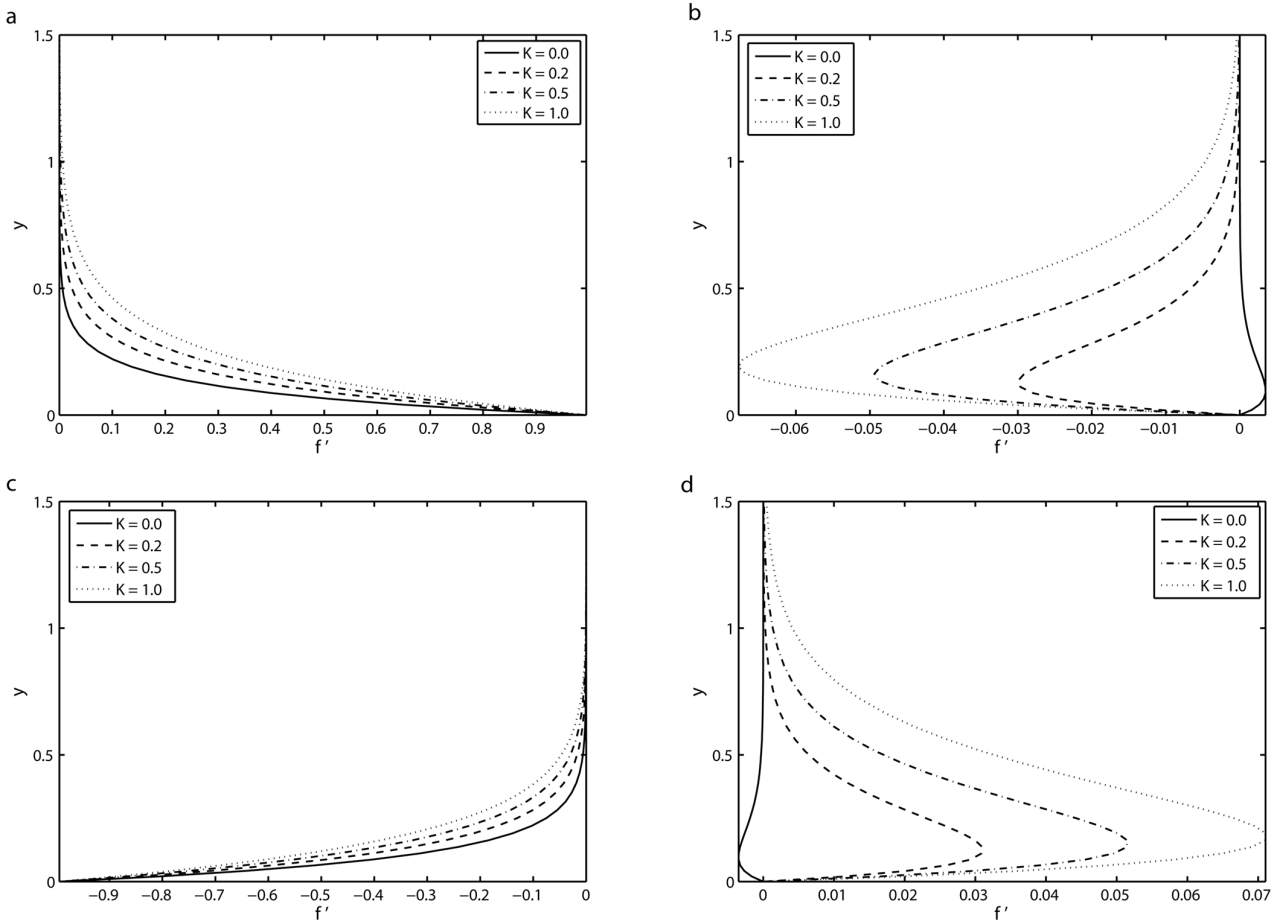
Transverse profiles of the velocity *f*′ for four different values of *K* for the fifth period *τ* ∈ [8*π*, 10*π*] for which a periodic velocity field has been reached: (*a*) *τ* = 8.5*π* (*b*) *τ* = 9*π* (*c*) *τ* = 9.5*π* and (*d*) *τ* = 10*π* with *S* = 2, *β* = 10 and *γ* = 0.5.

Fig 4 depicts the variation of the viscoelastic parameter *K* on the transverse profile of the velocity *f*′ for different values of *τ* = 8.5*π*, 9*π*, 9.5*π* and 10*π* in the fifth period *τ* ∈ [8*π*, 10*π*] for which a periodic motion has been reached. It can be seen from Fig 4(A) that at *τ* = 8.5*π*, the velocity *f*′ decays from unity at the surface to zero far away from the surface. It is also found that at this instant of time, there is no oscillation in the velocity and it is an increasing function of the viscoelastic parameter *K*, i.e. by increasing the values of *K*, the boundary layer becomes thicker. Fig 4(B) presents the velocity component *f*′ at time instant *τ* = 9*π* for various values of *K*. At this time instant, the velocity profile *f*′ is zero at the sheet (*y* = 0) and far away from the wall it again approaches to zero. It is also observed that near the surface, there exists some oscillations in the velocity field and the amplitude of the these oscillations increases with *K*. These oscillations in the transverse profile is an evidence of a phase shift in the viscoelastic fluid (*K* ≠ 0) against the viscous fluid (*K* = 0). Fig 4(C) and 4(D) display the velocity field *f*′ for others two time instants within the fifth periods. It is evident from Fig 4(C) and 4(D) that the flow in the whole flow domain is almost in phase with the sheet oscillations in the case of Newtonian fluid (*K* = 0), as shown from the solid lines displayed in Fig 4(A)–4(D). Furthermore, we can see from Fig 4 that the boundary layer thickness is increased by increasing the value of *K*.

**Fig 5:**
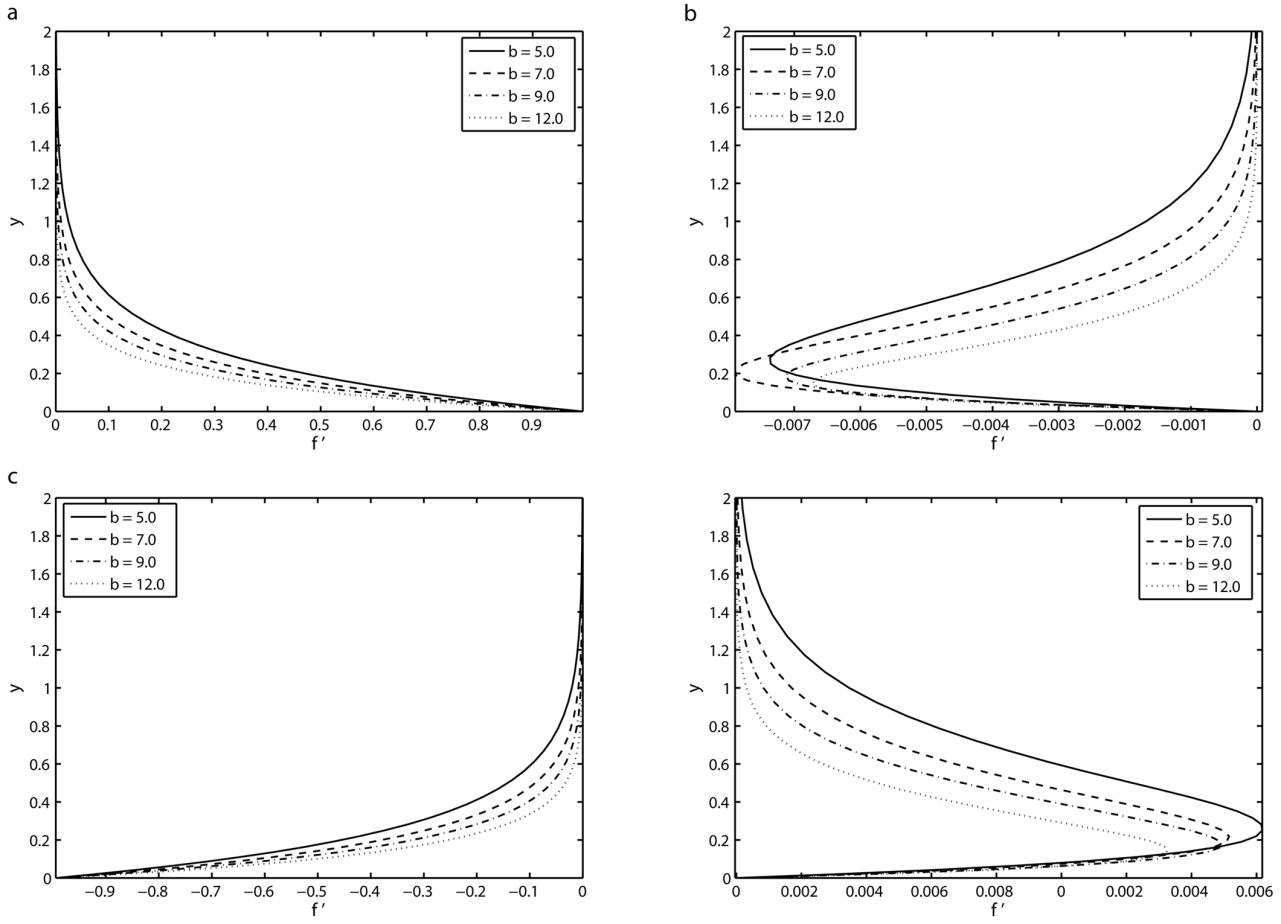
Transverse profiles of the velocity *f*′ for four different values of *β* in the fifth period *τ* ∈ [8*π*, 10*π*] for which a periodic velocity field has been reached (a) *τ* = 8.5*π* (b) *τ* = 9*π* (c) *τ* = 9.5*π* and (d) *τ* = 10*π* with *S* = 1, *K* = 0.2 and *γ* = 2.

Fig 5 illustrates the effect of the combined parameter *β* on the transverse profile of the velocity component *f*′ for *τ* = 8.5*π*, 9*π*, 9.5*π* and 10*π* with *S* = 1, *γ* = 2 and *K* = 0.2. It is evident from this figure that an increase in the Hartmann number or permeability parameter causes a reduction in the velocity field and the boundary layer thickness(Fig 5(A)). However, at *τ* = 9*π* (Fig 5(B) and *τ* = 10*π* (Fig 5(D)), there exist the oscillations with fairly small amplitudes in the transverse profiles of *f*′ near the wall. It is also noted that with increase in combined parameter *β*, the phase difference in *f*′ is almost invisible.

**Fig 6:**
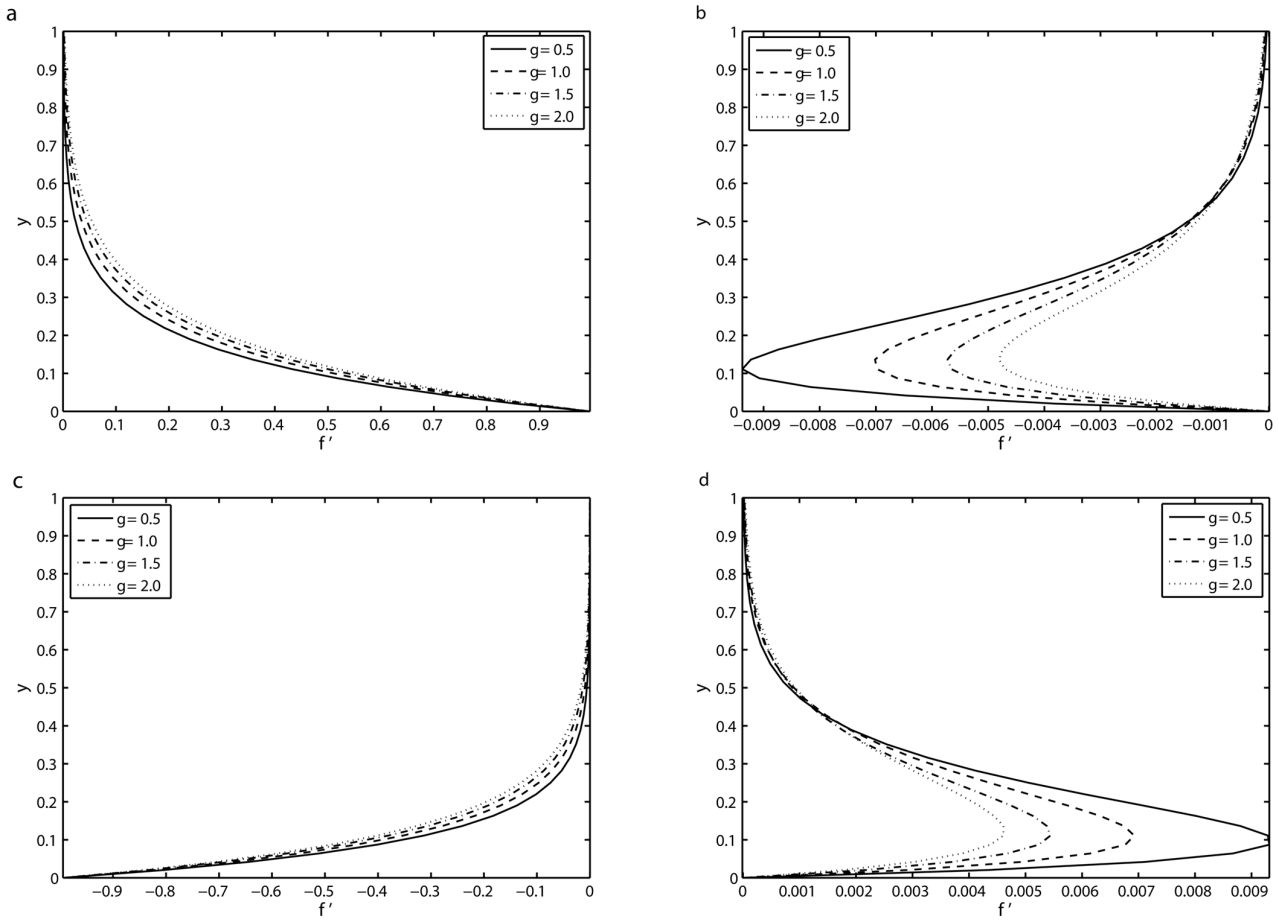
Transverses profile of the velocity *f*′ for four different values of *γ* in the fifth period *τ* ∈ [8*π*, 10*π*] for which a periodic velocity field has been reached: (*a*) *τ* = 8.5*π*, (*b*) *τ* = 9*π* (*c*) *τ* = 9.5*π* and (*d*) *τ* = 10*π* with *S* = 2, *K* = 0.1 and *β* = 10.

Fig 6 presents the variation in the transverse profile of the velocity field *f*′ for various values of mass suction/injection parameter *γ* at *τ* = 8.5*π*, 9*π*, 9.5*π* and 10*π* in the fifth period with *S* = 2, *β* = 10 and *K* = 0.1. The change in the velocity *f*′ for different values of *γ* at time *τ* = 8.5*π* can be seen from Fig 6(A). It is found that *f*′ = 1 at the sheet *y* = 0 and it approaches to zero far away from the sheet. Furthermore, the velocity profile is increased by increasing the value of the *γ*. The influence of *γ* on the velocity *f*′ at the time *τ* = 9*π* is presented in Fig 6(B). It is evident that at this time point the velocity takes zero value both at the wall and far away from the surface. The amplitude of oscillations near the plate decreases with increasing *γ*. The velocity fields for other two time points within the fifth are plotted in Fig 6(C) and 6(D) with the similar results as observed in Fig 6(A) and 6(B).

**Fig 7:**
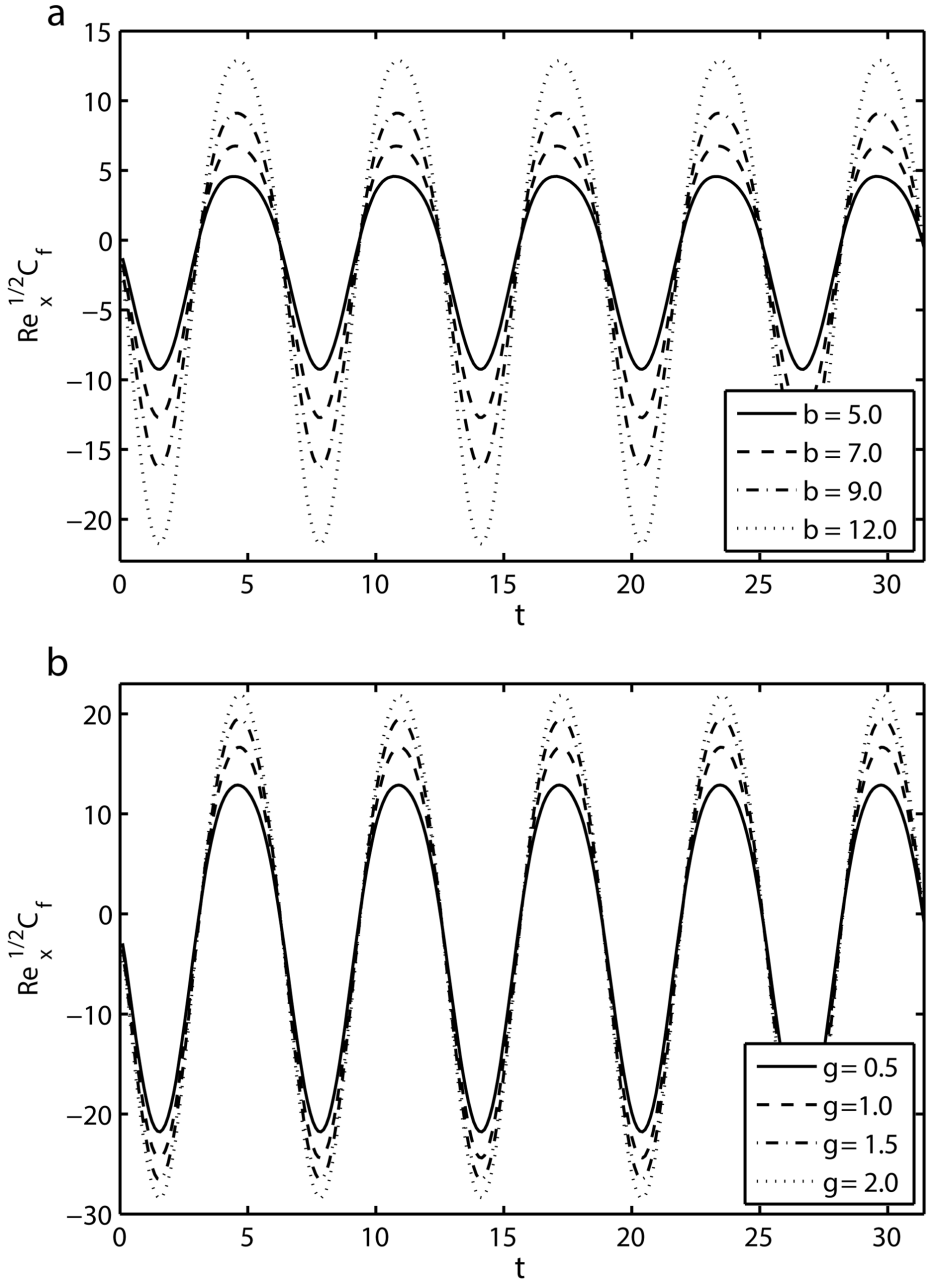
Time-series of the skin friction coefficient ${\text{Re}}_{x}^{1/2}C_{f}$ in the first five periods *τ* ∈ [8*π*, 10*π*]: (a) effects of *β* with *K* = 0.2, *S* = 1 and *γ* = 0.5 and (b) effects of *γ* with *K* = 0.2, *S* = 1 and *β* = 12.

Fig 7 shows the effects of the combined parameter *β* and the mass suction/injection parameter *γ* on the time-series of shear stress at the wall ${\text{Re}}_{x}^{1/2}C_{f}$ for the first five periods *τ* ∈ [0,10*π*]. Fig 7(A) gives the variation of the combined parameter *β* on the skin-friction coefficient ${\text{Re}}_{x}^{1/2}C_{f}.$ It is evident that skin friction is oscillatory in nature and amplitude of oscillations increases with increasing *β*. Fig 7(B) displays the effects of *γ* on the skin-friction coefficient ${\text{Re}}_{x}^{1/2}C_{f}$ with *S* = 1, *β* = 12 and *K* = 0.2. It is noted that the oscillation amplitude of the skin-friction coefficient ${\text{Re}}_{x}^{1/2}C_{f}$ increases for large values of mass suction/injection parameter *γ*.

**Fig 8:**
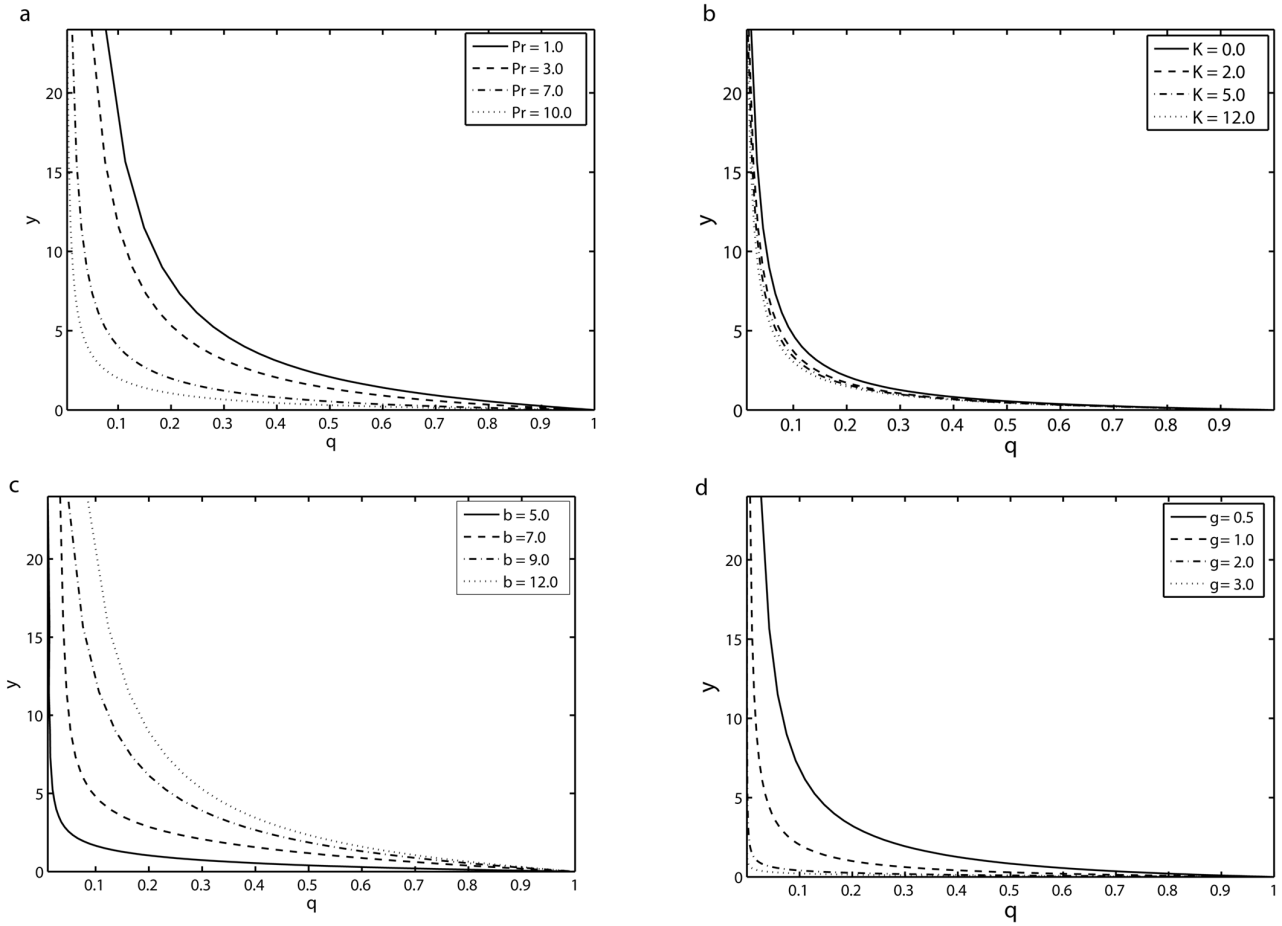
Transverse profiles of the temperature field *θ* at the time point *τ* = 8*π*: (a) effects of Pr with *K* = 0.2, *γ* = 0.5, *S* = 1, *β* = 10, (b) effects of *K* with *γ* = 1.5, *S* = 0.1, *β* = 12 and Pr = 1.5 and (*c*) effects *β* of with *K* = 0.2, *S* = 1, *γ* = 0.5 and Pr = 5 (d) effects of *γ* with *K* = 0.1, *S* = 2, *β* = 12 and Pr = 5.

Fig 8 displays the effect of the Prandtl number Pr, viscoelastic parameter *K*, combined parameter of magnetic field and permeability of porous medium *β* and the mass suction/injection parameter *γ* on the transverse profile of the temperature *θ* for the time point *τ* = 8*π*. Fig 8(A) shows the variation of the transverse profile of the temperature distribution *θ* for different values of Pr at the time point *τ* = 8*π*. This figure shows that thermal boundary layer thickness decreases with increasing Prandtl number. In fact the Prandtl number represents the ratio of momentum diffusivity to thermal diffusivity; larger values of Prandtl number correspond to fluids with weaker thermal diffusivity. Thus thermal boundary layer thickness in fluids with greater Prandtl number is small in comparison with fluids having lower Prandtl number. In view of above fact, it may be concluded that Prandtl number play a key role in cooling process. In another words it may be used to control the thickness of momentum and thermal boundary layers. Fig 8(B) depicts the transverse profiles of temperature *θ* for different values of viscoelastic parameter *K* for the time point *τ* = 8*π*. It is noticed from this figure that influence of viscoelastic parameter *K* is to decrease the temperature of the fluid. Fig 8(C) illustrates temperature profile *θ* for various values of *β* at the time point *τ* = 8*π* by keeping all other parameters fixed. It is observed that as we increase the values of *β*, both temperature *θ* and thermal boundary layer thickness are increased. The influence of the mass suction/injection parameter *γ* on the temperature field *θ* can be seen from Fig 8(D). It is found that from this figure that the temperature is a decreasing function of *γ*. The thermal boundary layer thickness also decreases by increasing *γ*.

**Fig 9:**
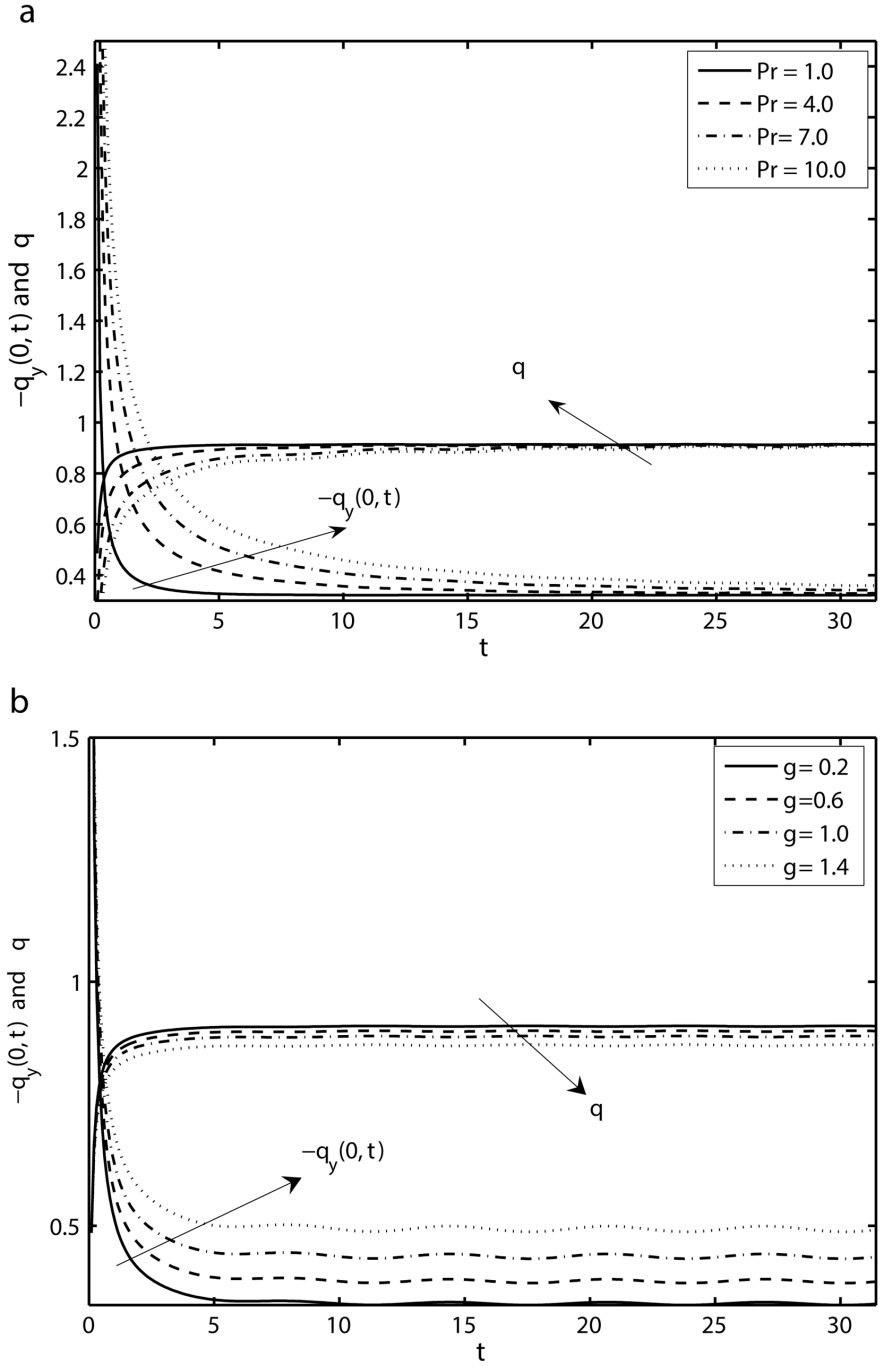
Time-series of the Nusselt number ${\text{Re}}_{x}^{-1/2}Nu_{x}$ and temperature profile *θ* in the first five periods *τ* ∈ [0, 10*π*] at a fixed distance from the sheet *y* = 0.25: (a) effects of Pr with *K* = 0.1, *γ* = 0.1, *β* = 12 and *S* = 1 and (b) effects of *γ* with *K* = 0.1, *β* = 12, and *S* = 2, Pr = 0.5.

Fig 9 presents the results by varying Prandtl number Pr and the mass suction/injection parameter *γ* on the time-series of the temperature distribution *θ* and the local Nusselt number ${\text{Re}}_{x}^{-1/2}Nu_{x}$ in the first five periods *τ* ∈ [0, 10*π*] at a fixed distance *y* = 0.25 from the sheet. From Fig 9(A), it can be seen that with the increase of Prandtl number Pr, i.e., with decrease of thermal diffusivity or the increase of momentum diffusivity, the fluid temperature decreases.

Fig 9(A) also shows that the magnitude of the local Nusselt number ${\text{Re}}_{x}^{-1/2}Nu_{x}$ is increased by increasing the values of Pr. Fig 9(B) illustrates the effects of the mass suction/injection parameter *γ* on the temperature profile *θ* and the local Nusselt number ${\text{Re}}_{x}^{-1/2}Nu_{x}$ in the first five periods *τ* ∈ [0, 10*π*]. It is noted from this figure that with the increase in the mass suction/injection parameter *γ*, the decrease in temperature *θ* with time becomes slower. Furthermore, a small oscillation, which is superimposed on the monotonically increasing temperature time-series, can be identified for large values of *γ*. It is further observed that local Nusselt number ${\text{Re}}_{x}^{-1/2}Nu_{x}$ increases with *γ*. A common observation from Fig 9(A) and 9(B) is that for *τ* = 0, the local Nusselt number attains a maximum and then decreases monotonically because of the given initial conditions i.e., the temperature gradient at the sheet has its maximum initially which decreases with time.

Table 1 shows the numerical values of the skin friction coefficient ${\text{Re}}_{x}^{1/2}C_{f}$ for various values of *S*, *K*, *β* and *γ* at *τ* = 1.5*π*, 5.5*π* and 9.5*π*. It is evident from this table that the values of skin friction coefficient for the three different time points *τ* = 1.5*π*, *τ* = 5.5*π* and *τ* = 9.5*π* are almost identical. Furthermore, we can see that the periodic motion may be reached within the first period when the initial conditions are set up. However, the change of the skin friction coefficient from positive to negative by increasing the value of *K* indicates the large phase difference as *K* increases. It is also noted that the value of the skin friction coefficient ${\text{Re}}_{x}^{1/2}C_{f}$ are increased as the relative frequency to the stretching rate *S*, combined parameter *β* and the mass suction/injection parameter *γ* are increased. Table 2 gives the numerical values of the local Nusselt number ${\text{Re}}_{x}^{-1/2}Nu_{x}$ for various values of the Prandtl number Pr, the viscoelastic parameter *K*, combined parameter of magnetic field and permeability of porous medium *β* and the mass suction/injection parameter *γ* at the four different times points *τ* = 2*π*, *τ* = 4*π*, *τ* = 6*π* and *τ* = 8*π*. It can be seen that the local Nusselt number increases by increasing the value of Pr, *K* and *γ* while it decreases by increasing *β* at all four times points *τ* = 2*π*, *τ* = 4*π*, *τ* = 6*π* and *τ* = 8*π*. Moreover, the values of local Nusselt number are also decrease when the time increases from *τ* = 2*π* to *τ* = 8*π* due to the decrease in the rate of heat transfer near the sheet.

**Table 1:** Numerical values of the skin-friction coefficient ${\text{Re}}_{x}^{1/2}C_{f}$ for different values of *γ*, *M*, *S*, *K* and *λ* at three different time points *τ* = 1.5*π*, 5.5*π* and 9.5*π*.

*S*	*K*	*β*	*γ*	*1*.*5π*	*5*.*5π*	*9*.*5π*
0.5	0.2	12.0	0.1	7.712793	7.712849	7.712781
1.0				7.824557	7.824703	7.824677
2.0				8.146671	8.147018	8.146691
3.0				8.570725	8.570726	8.570721
4.0				9.065895	9.066202	9.066481
1.0	0.0			12.182205	12.182195	12.182195
	0.2			7.824557	7.824703	7.824677
	0.5			1.846781	1.846511	1.846236
	0.8			-3.577653	-3.577136	-3.577650
	1.0			-6.938216	-6.938253	-6.938675
1.0	0.2	5.0		3.038148	3.038256	3.037955
		7.0		4.355440	4.355567	4.355471
		9.0		5.716519	5.716240	5.716628
		12.0		7.824557	7.824703	7.824677
		15.0		9.992332	9.992408	9.992349
		12.0	0.2	9.416449	9.416035	9.417047
			0.5	12.837937	12.839594	12.833545
			0.8	15.306652	15.294266	15.292671
			1.0	16.664039	16.656905	16.651919
			1.5	19.506594	19.486431	19.500553

**Table 2:** Numerical values of the local Nusselt number ${\text{Re}}_{x}^{-1/2}Nu_{x}$ for different values of Pr, *K*, *β* and *γ* at four different time points *τ* = 2*π*, 4*π*, 6*π* and 8*π* when *S* = 3.

*Pr*	*K*	*γ*	*β*	*2π*	*4π*	*6π*	*8π*
0.3	0.1	0.1	12.0	3.286656	3.250347	3.246147	3.245864
0.5				3.442377	3.300958	3.280489	3.279686
1.0				3.949524	3.528154	3.414912	3.381638
2.0				5.049508	4.140732	3.838137	3.707441
3.0				6.127671	4.814339	4.339281	4.116033
1.0	0.0		10.0	3.929795	3.510038	3.396825	3.363732
	0.3			3.957418	3.536146	3.420409	3.387667
	0.8			3.975992	3.554076	3.435345	3.403153
	1.0			3.979141	3.557149	3.437445	3.405464
	1.5			3.981102	3.559297	3.437308	3.405878
	0.2	0.0	12.0	3.735224	3.325657	3.215876	3.183788
		0.5		4.967091	4.498941	4.368397	4.331065
		1.0		6.513363	5.997575	5.848653	5.807043
		1.5		8.396179	7.850689	7.687753	7.643900
		1.8		9.694819	9.142083	8.973502	8.929473
		0.1	9.0	3.969607	3.546253	3.434544	3.400663
			12.0	3.967523	3.544466	3.432311	3.398531
			15.0	3.963304	3.540774	3.427898	3.394361
			18.0	3.956978	3.535232	3.421240	3.388076
			20.0	3.945905	3.525353	3.409733	3.376938

## Concluding Remarks

In present paper, we analyzed the flow and heat transfer of a viscoelastic fluid due to the oscillation of an infinite porous stretching sheet with magnetic field in a porous medium. A coordinate transformation is used to transform the semi-infinite flow domain to a finite computational domain and a suitable finite difference method is used to solve the governing partial differential equations. The time-series of the flow velocity, the temperature, the structure of the boundary layer near the plate for different values of involved parameters are graphically presented and discussed. The following observations may be made from the numerical results:

- The flow field generated by flat sheet which is suddenly stretched periodically rapidly becomes periodic, at most after three or four periods.
- The amplitude of velocity time-series is suppressed for large values of combined parameter. On the contrary, it increases with increasing the viscoelastic parameter.
- The flow exists only within a boundary layer near the plate, whilst the heat can be transferred to an infinitely large distance with the increase of time.
- The behavior of temperature is monotonic with time rather than oscillatory.
- The temperature and thermal boundary layer thickness increases with increasing combined parameter while converse trend is noted with increasing viscoelastic parameter and mass suction/injection parameter.
